# Prevalence and determinants of concurrent wasting and stunting and other indicators of malnutrition among children 6–59 months old in Kersa, Ethiopia

**DOI:** 10.1111/mcn.13172

**Published:** 2021-03-16

**Authors:** Aklilu Abrham Roba, Nega Assefa, Yadeta Dessie, Abebe Tolera, Kedir Teji, Hemler Elena, Lilia Bliznashka, Wafaie Fawzi

**Affiliations:** ^1^ College of Health and Medical Sciences Haramaya University Dire Dawa Ethiopia; ^2^ Harvard T.H. Chan School of Public Health Harvard University Boston Massachusetts USA

**Keywords:** concurrent wasting and stunting, Ethiopia, Kersa HDSS, point‐of‐use water treatment, stunting, underweight, WaSt, wasting

## Abstract

Malnutrition is the leading cause of poor child health in Ethiopia, and progress to avert it is unacceptably slow. In addition, little is known about the magnitude and factors associated with concurrent wasting and stunting (WaSt). Therefore, this study aimed to assess the prevalence and factors associated with WaSt, wasting, stunting and underweight among children 6–59 months in Kersa Health and Demographic Surveillance System, Ethiopia. Data from a total of 1091 children and their parents' were analysed from a cross‐sectional study. Household questionnaires and anthropometric measurements were used for data collection. Height‐for‐age, weight‐for‐height and weight‐for‐age indices are expressed as standard deviation units from the mean for the reference group. Multivariate analyses were conducted to identify factors associated with WaSt, wasting, stunting and underweight. Statistical significance was declared at *p* < 0.05. The prevalence of indicators of malnutrition was WaSt (5.8%), wasting (16.8%), stunting (53.9%) and underweight (36.9%). Children aged 6–17 months had a higher odds of wasting (adjusted odds ratio [aOR] 1.8, 95% confidence interval [CI] 1.12–2.75) compared with those aged 36–59 months, whereas children aged 18–35 months (aOR 2.4, 95% CI 1.65–3.47) and 36–59 months (aOR 1.6, 95% CI 1.07–2.37) had higher odds of stunting compared with those aged 6–17 months. Similarly, children aged 18–35 months (aOR 1.6, 95% CI 1.07–2.37) and 36–59 months (aOR 2.2, 95% CI 1.52–3.10) had higher odds of underweight compared with children aged 6–17 months. Households that did not treat drinking water at point of use were at higher odds of WaSt (aOR 3.3, 95% CI 1.16–9.27) and stunting (aOR 1.9, 95% CI 1.31–2.85) compared with those who did treat drinking water. Boys were more likely to be WaSt, wasted, stunted and underweight. Cough was associated with WaSt, wasting and underweight. Furthermore, maternal education, maternal occupation and maternal age were significantly associated with wasting. Maternal body mass index (BMI) of less than 18.5 kg/m^2^ and maternal BMI between 18.5 and 25 kg/m^2^ were associated with child stunting. In Kersa, the prevalence of WaSt, wasting, stunting and underweight is very high and requires urgent public health intervention. This study highlights point‐of‐use water treatment, maternal education, hygiene and sanitation, child health service utilization and maternal BMI as important areas to improve to target child malnutrition. Furthermore, a community‐based programmatic and policy direction for early identification and management of WaSt in addition to other indicators of malnutrition is recommended.

**Abbreviations**
aOR: adjusted odds ratio
cOR: crude odds ratio
EPHI: Ethiopian Public Health Institute
HAZ: height‐for‐age *z*‐scores
HDSS: Health and Demographic Surveillance System
WaSt: concurrent wasting and stunting
WAZ: weight‐for‐age *z*‐scores
WHZ: weight‐for‐height *z*‐scores

**Key messages**

- The prevalence of WaSt, wasting, stunting and underweight was 5.8%, 16.8%, 53.9% and 36.9%, respectively.
- WaSt was significantly associated with child's sex, cough and point‐of‐use treatment of drinking water.
- Wasting was significantly associated with child's age, child's sex, cough, maternal education, maternal occupation and maternal age.
- Stunting was significantly associated with child's age, water source, maternal BMI and point‐of‐use treatment of drinking water.
- Underweight was significantly associated with child's sex, child's age, cough, presence of toilet facility and point‐of‐use water treatment.

## 1 INTRODUCTION

Globally, children under 5 years of age face multiple burdens: 144 million are stunted, and 47 million are wasted. Africa (with 40% of children under 5 stunted and 27% wasted) and Asia (with 54% of children under 5 stunted and 69% wasted) bear the greatest share of all forms of malnutrition among under‐5 children (UNICEF et al., 2020). Malnutrition contributes to nearly half of all child deaths worldwide and to a significant burden of suboptimal development among surviving children (FAO et al., 2017).

While stunting in children under 5 years of age is declining globally, Africa is the only region where the number of stunted children rose from 2000 to 2019 (UNICEF et al., 2020). Results from the 2019 Ethiopian Mini Demographic and Health Survey (EMDHS) show that 37% of children under 5 years of age were stunted, 7% of were wasted and 21% were underweight in Ethiopia (Ethiopian Public Health Institute [EPHI], 2019). A recent systematic review showed that the overall pooled prevalence of stunting, underweight and wasting in Ethiopia was 42%, 33% and 15%, respectively (Abdulahi et al., 2017). However, regional disparities were observed. In Haramaya, in eastern Ethiopia, the prevalence of stunting, wasting and underweight among children under 5 years old was 45.8%, 10.7% and 21%, respectively (Yisak et al., 2015).

Several studies in Ethiopia noted predictors of child malnutrition including low parental education (Abdulahi et al., 2017; Asfaw et al., 2015; Dessie et al., 2019; EPHI, 2019), low household economic status (Dessie et al., 2019; Motbainor & Taye, 2019), large family size or overcrowding (Yisak et al., 2015), having two or more under‐5 children and lack of maternal access to a health facility (Egata et al., 2014). Among child‐related factors, male sex (Asfaw et al., 2015; Kasaye et al., 2019; Yisak et al., 2015), diarrheal diseases (Asfaw et al., 2015; Kasaye et al., 2019; Yisak et al., 2015), fever (Yisak et al., 2015) and age of the child (above 12 months) (Kasaye et al., 2019) were associated with increased stunting and underweight in Ethiopia.

Concurrent wasting and stunting (WaSt) is the coexistence of wasting and stunting at the same time in a child (Garenne et al., 2019). Joint global or regional estimates for these combined conditions are limited (UNICEF et al., 2020). A meta‐analysis of data from 84 countries indicated that the pooled prevalence of WaSt was 3% (95% confidence interval [CI] 2.97–3.06) with the highest burden in Africa (3.5%), followed by Asia (3.4%) (Khara et al., 2018). Another sub‐Saharan African report shows that the prevalence of WaSt was 5% in Uganda (Odei Obeng‐Amoako et al., 2021) and 6.2% in Senegal (Garenne et al., 2019). Evidence indicates that children with WaSt have high mortality risk (Garenne et al., 2019; Khara et al., 2018) and are likely to have dehydrating diarrhoea (Nuzhat et al., 2020).

There is limited knowledge on the magnitude of and factors associated with WaSt in Ethiopia. Similarly, factors that may affect wasting, stunting, underweight and WaSt simultaneously need further investigation. Thus, we assessed the prevalence and factors associated with WaSt, wasting, stunting and underweight among children under 5 years of age in Kersa, Ethiopia.

## 2 MATERIALS AND METHODS

### 2.1 Study setting

This study was conducted in the Kersa Health and Demographic Surveillance System (HDSS) site in the Oromia region of eastern Ethiopia. The Kersa district has 38 kebeles (the smallest administrative unit in Ethiopia), of which 24 were included in the HDSS (three are urban and 21 are rural). The 2007 national census reported a total population for this district of 170 816, composed of 86 134 men and 84 682 women. A total of 11 387 or 6.7% of the district's population were urban dwellers, 97% were Muslim and 96.25% were Oromo by ethnicity (CSA, 2007). Most inhabitants are farmers, with a minority working in small trade, government posts or casual labourers. During the establishment of the research site, kebeles were selected to provide representation from the three agroecological zones (highland, midland and lowland), as well as urban and rural areas. According to the 2019 EMDHS, 35.6%, 4.7% and 16.1% of under‐5 children were stunted, wasted and underweight (< −2 SD for each measurement), respectively, in Oromia regional state (EPHI, 2019).

### 2.2 Study design

We conducted a cross‐sectional quantitative survey among under‐5 children in 12 kebeles in the Kersa HDSS from June to September 2019.

### 2.3 Sample size and sampling technique

A total of 1200 households from 12 kebeles (100 under‐5 children from each kebele) were selected from the Kersa HDSS database using simple random sampling. Children were eligible to be selected from the database if they were aged 6–59 months, residing in one of the 12 selected kebeles, and had a living mother and father. Only one 6‐ to 59‐month‐old child (index child) was selected from each household. The data extracted from the database included location ID, household ID and individual ID; name, age and sex of a child; and full names of the father and mother. It also included the names of gendas (subsections of kebeles) in the selected kebeles.

### 2.4 Data collection and measurements

Household questionnaires and anthropometric measurements were used for data collection. Anthropometry was directly assessed for all adult female respondents. Height was measured to the nearest 0.1 cm with the subject barefoot, using a stadiometer. Child weight was measured to the nearest 0.1 kg using a standard clinical scale set at the tar mother–child function. Before weighing, the diaper was removed and the child was stripped to minimal clothing. For children below 24 months, the length was measured using a length mat, and for those 24–59 months, standing height was measured using a stadiometer to the nearest 0.1 cm. Child mid‐upper arm circumference (MUAC) was measured to the nearest 0.1 cm using nonstretchable measuring tape from the left arm. The measurement was taken at the midpoint between the elbow and the shoulder bones of the non‐dominant arm with the arm relaxed and hanging down the side of the body. Bilateral pitting oedema was not assessed based on the assumption that its prevalence would be too low in this community‐based study (Bilukha & Leidman, 2018).

### 2.5 Variables

The dependent variables were WaSt, wasting, stunting and underweight.

Parental sociodemographic characteristics (maternal age, maternal education, maternal occupation, paternal education and paternal occupation), maternal body mass index (BMI) status, availability and type of toilet, water sources and water treatment at point of use, and child health status in the 2 weeks before data collection were included as independent variables.

Wasting (acute malnutrition) was defined as weight‐for‐height *z*‐scores (WHZ) −2 SD below the mean of the reference population. Wasting was also defined using MUAC less than 12.5 cm. Stunting (chronic malnutrition) was defined as height‐for‐age *z*‐scores (HAZ) less than 2 SD below the mean (−2 SD) of the reference population. Underweight was defined as weight‐for‐age *z*‐scores (WAZ) at 2 SD or more below the mean (−2 SD) of the reference population (World Health Organization [WHO], 2006). A child was defined as WaSt if he or she was wasted and stunted at the same time (Myatt et al., 2019). Moderate and severe malnutrition were classified as follows: moderately stunted, HAZ < −2·0; moderately underweight, WAZ < −2·0; moderately wasted, WHZ < −2·0 or MUAC 11.5 to 12.5 cm; severely stunted, HAZ < −3·0; severely underweight, WAZ < −3·0; severely wasted, WHZ < −3·0 or MUAC < 11.5 cm; and WaSt, WHZ < −2·0 and HAZ < −2·0 (WHO, 2006).

Maternal BMI was defined as weight in kilograms divided by the square of height in metres. Underweight was defined as a BMI of less than 18.5 kg/m^2^, overweight and obese as a BMI greater than or equal to 25 kg/m^2^ and normal BMI as greater than or equal to 18.5 kg/m^2^ and less than 25 kg/m^2^ (Institute of Medicine [IOM], 2005).

Educational status of the mother or father was classified as ‘no formal education’ when he/she responded unable to read/write or only read/write whereas attending/completed colleges/university education was regarded as ‘higher level education’); occupation was classified as farmer or nonfarmer, and point‐of‐use water treatment was classified as chlorinate, boil or do nothing.

Point‐of‐use drinking water treatment was categorized into chlorination, boiling and did nothing based on the most commonly used methods in Kersa.

### 2.6 Data processing and analysis

Child weight, height/length, age, sex, MUAC and ID data were exported to ENA for SMART version 2020 (ENA for SMART, 2020) for calculations of *z*‐scores using the WHO (2006) Child Growth Standards, HAZ, WAZ and WHZ, and imported into SPSS Version 25 (IBM Corp, 2017) for final analysis. All WHO flags were excluded from further analysis (WHZ −5 to 5; HAZ −6 to 6; and WAZ −6 to 5). Then data were recoded again based on the *z*‐scores for wasting, stunting, underweight and WaSt. MUAC values less than 7 cm and greater than 22 cm were excluded as outliers (Leidman et al., 2019).

Bivariate analysis was conducted to examine the association between dependent and independent variables; crude odds ratios (cORs) and their 95% CIs were calculated. All variables that had a *p*‐value less than 0.2 in the bivariate analysis were included in the multivariate binary logistic regression model to identify independent variables that were associated with the stunting, wasting, underweight and WaSt using SPSS Version 25. All logistic regression assumptions (adequacy of sample size in each category, multicollinearity and outliers) were met (Pallant, 2010). The Hosmer–Lemeshow goodness‐of‐fit test had a significance value of greater than 0.05 for all wasting, stunting, underweight and WaSt, indicating good fit of the model. Similarly, the Omnibus Tests of Model Coefficients was less than 0.005, indicating good fit of the model (Pallant, 2010). Multicollinearity was tested among the independent variables by using the variance inflation factor (VIF) and the tolerance test. The result of the VIF ranged from 1.005 to 1.952 (expected to be less than 10) whereas the tolerance test was less than one and greater than 0.1, which was within the normal limit (Pallant, 2010).

### 2.7 Ethical considerations

The study was conducted after securing ethical clearance from Haramaya University (IHRERC), the National Research Ethics Review Committee of Ethiopia (SHE/SIM/144/708/19) and the Harvard T.H. Chan School of Public Health. Participation in the study was voluntary, and informed written consent was obtained from each parent of a child.

## 3 RESULTS

A total of 1091 households were included in this study with a response rate of 91%.

### 3.1 Parent's and household characteristics

Around half of the mothers (53.2%) and fathers (49.8%) had never received formal education; 80.8% of mothers and 84.9% of fathers were farmers. Regarding water source, only 486 (44.5%) households use piped/clean water. In less than half of the study households, water was treated at point of use before consumption (22.5% boiled and 25.3% chlorinated). A toilet was available only in 600 (55%) households, and open pit was the most common toilet type. Regarding maternal nutritional status, 166 (16.6%) mothers were underweight. Furthermore, 91 (8.3%) households had more than one under‐5 children (Table 1).

**TABLE 1:** Sociodemographic and household characteristics of parents in Kersa, 2019

Variable	Characteristics	Frequency (*n* = 1091)	Percentage
Maternal highest level of education	No formal education	580	53.2
	Completed high school	445	40.8
	Higher level education	66	6
Paternal highest level of education	No formal education	543	49.8
	Completed high school	458	42
	Higher level education	90	8.2
Maternal occupation	Agricultural	882	80.8
	Nonagricultural	209	19.2
Paternal occupation	Agriculture	926	84.9
	Nonagricultural	165	15.1
Water source	Piped, clean water	486	44.5
	Other nonpiped sources	605	55.5
Point‐of‐use water treatment	Do nothing	570	52.2
	Boil	245	22.5
	Chlorinate	276	25.3
Type of toilet	No	491	45
	Open pit	409	37.5
	Improved pit	13	1.2
	Flushed all type	57	5.2
	Others	121	11.1
Maternal BMI (1001)	Underweight	166	16.6
	Normal	740	73.9
	Overweight and obese	76	7.5

Abbreviation: BMI, body mass index.

### 3.2 Child characteristics and nutritional status

A slightly higher number of boys (54.4%) were included in the study than girls (45.6%). The mean child age was 31.9 ± 16.5 months (95% CI 30.9–32.9), and the median and mode were 32 and 24 months, respectively. In the preceding 2 weeks before data collection, the prevalence of cough, diarrhoea, fever and vomiting was 10%, 5%, 9% and 5%, respectively. After excluding outliers, we included 1001, 903, 1075 and 847 children in the analyses of wasting, stunting, underweight and WaSt, respectively. The prevalence of indicators of malnutrition was WaSt (5.8%), wasting (16.8%), stunting (53.9%) and underweight (36.9%) (Table 2).

**TABLE 2:** Health and nutritional status of 6‐ to 59‐month‐old children in Kersa, 2019

Variable	Characteristics	Frequency (*n* = 1091)	Percentage
Diarrhoea in the last 2 weeks	Yes	54	4.9
Cough in the last 2 weeks	Yes	109	10.0
Fever in the last 2 weeks	Yes	95	8.7
Vomiting in the last 2 weeks	Yes	55	5.0
Wasting by WHZ (*n* = 1001)	< −3 SD	107	10.7
	≥ −3 to < −2 SD	61	6.1
Wasting by MUAC (*n* = 803)	<11.5 cm	42	5.2
	11.5–12.5 cm	114	14.2
Stunting (*n* = 903)	< −3 SD	340	37.7
	≥ −3 to < −2 SD	147	16.3
Underweight (*n* = 1075)	< −3 SD	236	22.0
	≥ −3 to < −2 SD	161	15.0
WaSt (*n* = 847)	< −2 SD HAZ and WHZ	49	5.8

Abbreviations: HAZ, height‐for‐age *z*‐scores; MUAC, mid‐upper arm circumference; WaSt, concurrent wasting and stunting; WHZ, weight‐for‐height *z*‐scores.

### 3.3 Prevalence of nutritional status

**FIGURE 1:**
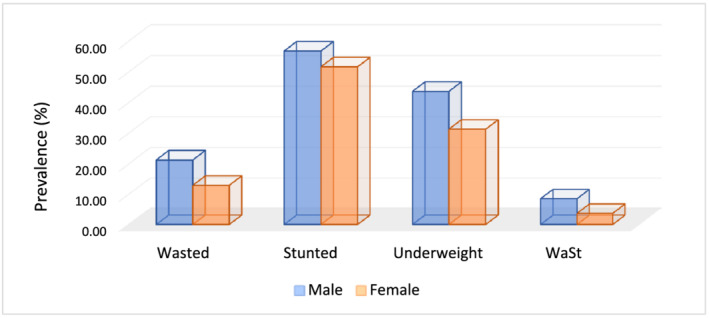
Nutritional status of under‐5 children based on sex in Kersa, Ethiopia, 2019. WaSt, concurrent wasting and stunting

A total of 16.8% (95% CI 14.7–19.4) of children were wasted with higher prevalence among boys than girls: 21% (95% CI 18–25) versus 13.1% (95% CI 10–16), respectively. Using MUAC, the prevalence of wasting was 19.4% with 5.2% severe and 14.2% moderate wasting. Severe wasting was most prevalent among children aged 6–17 months in both parameters (MUAC and WHZ) (Figure 1).

The prevalence of stunting was 53.9% (95% CI 50.1–56.7). The prevalence of severe stunting was 36.9% (95% CI 33.8–40.2), and the prevalence of moderate stunting was 16.5% (95% CI 14.1–19.1). Boys were more likely to be stunted (56.5%, 95% CI 51.5–61.5) than girls (51%, 95% CI 46.6–55.4). Severe stunting was also more prevalent among boys than girls. In contrast, moderate stunting was more common among girls than boys (18.2% vs. 14.1%, respectively).

The prevalence of underweight was 36.9% (95% CI 32.2–38.9). Severe underweight (21.9%) was more common than moderate underweight (15.1%). Similar to wasting and stunting, the underweight prevalence was higher among boys (45.9%) than girls (32.8%). Severe underweight was more prevalent among older children.

The prevalence of WaSt was 5.8% (95% CI 4.3–7.58). WaSt was more prevalent in boys than girls: 9% (95% CI 6–11) versus 4% (95% CI 2–5).

### 3.4 The age pattern of malnutrition

**FIGURE 2:**
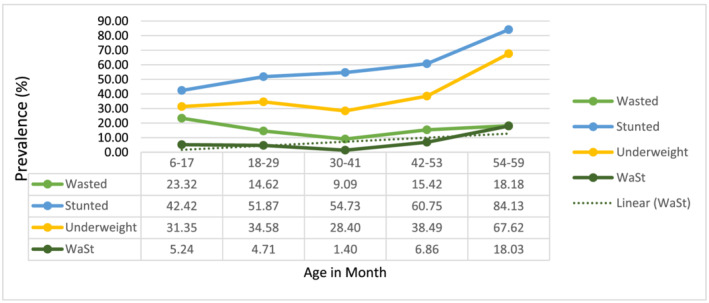
Age patterns of malnutrition among under‐5 children based on age in Kersa, Ethiopia, 2019. WaSt, concurrent wasting and stunting

The age patterns of wasting, stunting, underweight and WaSt were displayed in Figure 2. The proportion of wasted children declined rapidly from 23.1% (6–17 months) to 14.6% (18–29 months) and reached to the lowest level of 9.0% (30–41 months), and then slowly increased to 15.4% (42–53 months) and again increased to 18.2% at 54–59 months. The proportion of stunted children increased slowly with age, without any decline, and then reached a peak of 84.1% at 54–59 months. The proportion of underweight children slowly increased (from 31.4% at 6–17 months to 34.6% at 18–29 months) and then decreased (28.4% at 30–41 months) and began to increase and reached the peak at 54–59 months. The proportion of WaSt children had similar patterns with that of a wasted child with a fast increase from 6 to 17 months, reached to the lowest level at 30–41 months and then slowly reached a peak of 18.0% at 54–59 months. In children aged 54–59 months, all of the above‐mentioned malnutrition types reached a peak (except wasting) (Figure 2).

### 3.5 Factors associated with child wasting

Factors associated with child wasting using WHZ included male sex, younger age, maternal occupation, maternal education, maternal age and cough. Boys had higher odds of being wasted (adjusted odds ratio [aOR] 1.9, 95% CI 1.34–2.68) than girls, as did children aged 6–17 months (aOR 1.8, 95% CI 1.12–2.75) compared with children aged 36–59 months. Children whose mothers were farmers (aOR 0.4, 95% CI 0.21–0.69) had lower odds of wasting than nonfarmers, and children whose mothers had no formal education (aOR 2.6, 95% CI 1.43–4.53) had higher odds of wasting compared with children whose mothers attended formal education. Maternal age was positively associated with child's odds of wasting (aOR 1.0, 95% CI 1.00–1.06). Concerning health status, children with cough had 1.7 times higher odds of being wasted (aOR 1.7, 95% CI 1.01–2.99) than children with no cough (Table 3).

**TABLE 3:** Factors associated with wasting among children 6–59 months old in Kersa, 2019

Variables		cOR (95% CI)	*p*‐value	aOR (95% CI)	*p*‐value
Child age (in months)	6–17	1.8 (1.27–2.68)**	0.001	1.8 (1.12–2.75)**	0.014
	18–35	1.0 (0.65–1.58)	0.97	1.1 (0.69–1.79)	0.665
	36–59	1		1	1
Sex	Male	1.8 (1.30–2.54)*	0.001	1.9 (1.34–2.68)**	0.000
	Female	1		1	1
Other U5 child	Yes	0.5 (0.31–0.83)*	0.007	0.7 (0.38–1.22)	0.20
	No	1		1	
Cough in the last 2 weeks	Yes	0.7 (0.40–1.13)	0.132	1.7 (1.01–2.99)**	0.05
	No	1		1	
Maternal education	No formal	0.8 (0.57–1.12)	0.31	2.6 (1.43–4.53)**	0.001
	Formal	1		1	
Maternal occupation	Farmer	0.5 (0.32–0.87)*	0.011	0.4 (0.21–0.69)**	0.001
	Nonfarmer	1		1	0.148
Paternal education	No formal	0.9 (0.65–1.28)	0.446	0.7 (0.36–1.56)	0.14
	Formal	1		1	0.190
Maternal age	Maternal age	1.0 (0.98–1.04)	0.54	1.0 (1.004–1.06)**	0.028
Maternal BMI	Underweight	0.9 (0.42–1.75)	0.68	0.6 (0.30–1.26)	0.236
	Normal	0.9 (0.48–1.63)	0.69	0.7 (0.34–1.26)	0.20
	Overweight and obese	1		1	
Point‐of‐use treatment of drinking water	Nothing	1.3 (0.86–1.95)	0.22	0.9 (0.55–1.50)	0.712
	Boiling	1.3 (0.82–2.19)	0.25	0.9 (0.54–1.63)	0.813
	Chlorination	1		1	

Abbreviations: aOR, adjusted odds ratio; BMI, body mass index; CI, confidence interval; cOR, crude odds ratio; U5, under‐5.

* Denotes significant association in the binary logistic regression analysis.

** Denotes statistically significant association in the multivariable analysis, *p*‐values less than 0.05.

### 3.6 Factors associated with child stunting

Children aged 18–59 months, maternal BMI, water source and point‐of‐use water treatment were factors associated with stunting. Children aged 36–59 months (aOR 1.6, 95% CI 1.07–2.37) and 18–35 months (aOR 2.4, 95% CI 1.65–3.47) were at higher odds of stunting. Households that used nonpiped water and that did not treat drinking water were also at higher odds of stunting (aOR 1.5, 95% CI 1.07–2.00, and aOR 1.9, 95% CI 1.31–2.85, respectively). Stunting was also significantly associated with maternal BMI of less than 18.5 kg/m^2^ (underweight) (aOR 2.5, 95% CI 1.32–4.72) and BMI of 18.5–24.9 kg/m^2^ (normal) (aOR 1.9, 95% CI 1.08–3.28) (Table 4).

**TABLE 4:** Factors associated with stunting among children 6–59 months old in Kersa, 2019

Variables		cOR (95% CI)	*p*‐value	aOR (95% CI)	*p*‐value
Child age (in months)	36–59	1.5 (1.07–2.16)*	0.02	1.6 (1.07–2.37)**	0.022
	18–35	2.2 (1.62–3.05)*	0.000	2.4 (1.65–3.47)**	0.000
	6–17	1		1	
Sex	Male	1.2 (0.94–1.61)	0.125	0.8 (0.59–1.04)	0.094
	Female	1		1	
Other U5 child	None	1.6 (0.99–2.58)	0.056	1.6 (0.90–2.79)	0.108
	Yes	1		1	
Maternal education	No formal	0.9 (0.65–1.11)	0.24	1.0 (0.64–1.57)	0.61
	Formal	1		1	
Maternal occupation	Farmer	1.6 (1.11–2.15)*	0.01	1.2 (0.75–1.82)	0.49
	Nonfarmer	1		1	
Paternal education	No formal	0.9 (0.66–1.12)	0.26	0.8 (0.51–1.26)	0.335
	Formal	1		1	
Maternal age	Maternal age	1.0 (0.98–1.02)	0.63	1.0 (0.97–1.01)	0.401
Maternal BMI (kg/m^2^)	Underweight	2.9 (1.57–5.17)*	0.001	2.5 (1.32–4.72)**	0.005
	Normal	2.2 (1.32–3.74)*	0.003	1.9 (1.08–3.28)**	0.026
	Overweight and obese	1		1	
Water source	Nonpiped	1.7 (1.29–2.20)*	0.000	1.5 (1.07–2.00)**	0.017
	Piped clean	1		1	
Point‐of‐use treatment of drinking water	Nothing	1.9 (1.43–2.64)*	0.000	1.9 (1.31–2.85)**	0.001
	Boiling	1.1 (0.78–1.64)	0.511	1.1 (0.73–1.69)	0.622
	Chlorination	1		1	

Abbreviations: aOR, adjusted odds ratio; BMI, body mass index; CI, confidence interval; cOR, crude odds ratio; U5, under‐5.

* Denotes significant association in the binary logistic regression analysis.

** Denotes statistically significant association in the multivariable analysis, *p*‐values less than 0.05.

### 3.7 Factors associated with WaSt

Absence of cough in the 2 weeks prior to data collection was a protective factor for WaSt (aOR 0.3, 95% CI 0.13–0.66). Boys had two times the odds of WaSt compared with girls (aOR 2.3, 95% CI 0.27–4.39). On the other hand, children from households that did not treat drinking water at point of use had three times higher odds of WaSt than those who treated water with chorine (aOR 3.3, 95% CI 1.16–9.27) (Table 5).

**TABLE 5:** Factors associated with concurrent wasting and stunting among 6‐ to 59‐month‐old children in Kersa, 2019

Variables		cOR (95% CI)	*p*‐value	aOR (95% CI)	*p*‐value
Child age (in months)	36–59	0.8 (0.34–1.94)	0.64	1.5 (0.56–3.98)	0.42
	18–35	0.4 (0.68–2.66)	0.39	1.8 (0.80–4.23)	0.15
	6–17	1			
Sex	Male	2.4 (1.34–4.42)*	0.004	2.3 (1.271–4.39)**	0.006
	Female	1		1	
Other U5 child	None	0.5 (0.23–1.21)	0.132	0.5 (0.18–1.51)	0.23
	Yes	1		1	
Cough in the last 2 weeks preceding data collection	No	0.4 (0.18–0.80)*	0.01	0.3 (0.13–0.66)**	0.003
	Yes	1		1	
Maternal education	No formal	1.3 (0.69–2.3)	0.46	0.8 (0.30–2.05)	0.63
	Formal	1		1	
Maternal occupation	Farmer	13.0 (1.78–94.82)*	0.011	8.9 (0.71–112.51)	0.09
	Nonfarmer	1		1	
Paternal education	No formal	1.6 (0.86–2.87)	0.15	1.0 (0.38–2.59)	0.99
	Formal	1		1	
Paternal occupation	Farmer	10.2 (1.4–74.6)*	0.022	1.6 (0.13–19.79)	0.722
	Nonfarmer	1		1	
Maternal BMI	Underweight	4.8 (0.61–38.69)	0.14	2.0 (0.23–16.42)	0.54
	Normal	4.0 (0.54–29.49)	0.18	1.8 (0.23–13.61)	0.59
	Overweight and obese	1		1	
Water source	Nonpiped	1.1 (0.60–1.95)	0.79	0.6 (0.31–1.14)	0.12
	Piped clean	1		1	
Point‐of‐use treatment of drinking water	Nothing	4.7 (1.80–12.01)*	0.001	3.3 (1.16–9.27)**	0.025
	Boil	1.9 (0.58–5.99)	0.29	1.3 (0.37–4.27)	0.709
	Use chlorine	1		1	

Abbreviations: aOR, adjusted odds ratio; BMI, body mass index; CI, confidence interval; cOR, crude odds ratio; U5, under‐5.

* Denotes significant association in the binary logistic regression analysis.

** Denotes statistically significant association in the multivariable analysis, *p*‐values less than 0.05.

### 3.8 Factors associated with underweight

Factors associated with underweight included male sex, older age, cough in the last 2 weeks, presence of toilet facility and point‐of‐use water treatment. Male sex (aOR 1.8, 95% CI 1.35–2.26), cough in the preceding 2 weeks of data collection (aOR 2.0, 95% CI 1.29–2.97), age 36–59 months (aOR 2.2, 95% CI 1.52–3.10) and age 18–35 months (aOR 1.6, 95% CI 1.07–2.37) were associated with higher odds of underweight. Similarly, children from households that did not have a toilet or treat drinking water at point of use were at higher odds of underweight (Table 6).

**TABLE 6:** Factors associated with underweight among children 6–59 months old in Kersa, 2019

Variables		cOR (95% CI)	*p*‐value	aOR (95% CI)	*p*‐value
Child age (in months)	36–59	1.8 (1.30–2.40)*	0.000	2.2 (1.52–3.10)**	0.000
	18–35	1.3 (0.88–1.80)	0.213	1.6 (1.07–2.37)**	0.022
	6–17	1		1	
Sex	Male	1.8 (1.36–2.24)*	0.000	1.8 (1.35–2.26)**	0.000
	Female	1		1	
Cough in the last 2 weeks	Yes	1.8 (1.19–2.65)*	0.005	2.0 (1.29–2.97)**	0.002
	No	1		1	
Maternal age	Maternal age	1.0 (0.97–1.01)	0.44	0.9 (0.96–1.00)	0.117
Maternal education	No formal	0.9 (0.683–1.122)	0.292	1.0 (0.76–1.37)	0.89
	Formal	1		1	
Other U5 child	No	1.0 (0.618–1.509)	0.877	0.7 (0.39–1.10)	0.108
	Yes	1		1	
Toilet	No	1.4 (1.076–1.771)*	0.011	1.3 (1.01–1.72)**	0.041
	Yes	1		1	
Point‐of‐use treatment of drinking water	Nothing	1.5 (1.111–2.041)*	0.008	1.6 (1.09–2.21)**	0.026
	Boil	1.1 (0.768–1.604)	0.579	1.3 (0.88–1.92)	0.191
	Use chlorine	1		1	

Abbreviations: aOR, adjusted odds ratio; CI, confidence interval; cOR, crude odds ratio; U5, under‐5.

* Denotes significant association in the binary logistic regression analysis.

** Denotes statistically significant association in the multivariable analysis, *p*‐values less than 0.05.

## 4 DISCUSSION

This study found a very high prevalence of wasting and stunting in Kersa, Ethiopia, according to the WHO–UNICEF Technical Advisory Group on Nutrition Monitoring report prevalence thresholds for wasting (≥15) and stunting (≥30) (UNICEF et al., 2020). Children less than 18 months were more prone to be wasted, whereas children older than 18 months were stunted or underweight. Boys were more likely to be affected by all forms of malnutrition. Cough in the past 2 weeks was a determinant factor for wasting and underweight. Not treating water at point of use was a determinant factor for stunting, underweight and WaSt.

The prevalence of wasting in Kersa (16.8%) is comparable with that in East Belesa District (16%) (Fentahun et al., 2016) but lower than in Dabat District, in north‐west Ethiopia (18.2%) (Tariku et al., 2017) and in Shinille Woreda, eastern Ethiopia (20%) (Ma'alin et al., 2016). However, it is much higher than the national prevalence of 7% found in the EDHS, 2019 (EPHI, 2019) and 4.7% in Oromia regional state. We found a higher prevalence of wasting in Kersa compared with similar Ethiopian studies that found prevalence ranging from 7.1% to 13.4% (Abera et al., 2017; Asfaw et al., 2015; Ejigu et al., 2017; Gizaw et al., 2018; Motbainor & Taye, 2019; Tufa et al., 2018; Wasihun et al., 2018; Yisak et al., 2015). The high prevalence of wasting in Kersa may be attributable to persistent drought in the area, data collection season (preharvest) and the fact that more than half of the parents had never attended formal education and three fourths were farmers.

The prevalence of stunting in Kersa was 53.9%, of which 41% were severely stunted with very high public health significance. The overall prevalence of stunting is comparable with Tigray (49.2%) (Gebru et al., 2019), Arba Minch (47.9%) (Bogale et al., 2020), Bule Hora (47.6%) (Asfaw et al., 2015) and East Belesa District (57.7%) (Fentahun et al., 2016). However, it is much higher than the national average of 37% in 2019 (EPHI, 2019) and 33.4% in Shinille Woreda (Ma'alin et al., 2016).

In Kersa district, 5.8% of children were WaSt. This is comparable with 6.2% in Senegal (Garenne et al., 2019) and 4.9% in Uganda (Odei Obeng‐Amoako, et al., 2020) but higher than 1.4% reported in a Ghanaian study (Saaka & Galaa, 2016). Studies indicate that WaSt was more prevalent among boys (Odei Obeng‐Amoako, et al., 2021; Saaka & Galaa, 2016) and found to be a strong risk factor for child mortality (Garenne et al., 2019; Myatt et al., 2019). There is limited evidence on the burden of this condition, and it is an element that is worth pursuing in future work.

The present study also identified factors associated with WaSt, wasting, stunting and underweight. Being male, having a cough in the previous 2 weeks prior to the study and not treating drinking water at point of use were significantly associated with WaSt. Similarly, age of 6–17 months, male sex, having a cough in the past 2 weeks, no formal maternal education, maternal occupation of nonfarming and maternal age were positively associated with wasting. Also, stunting was significantly associated with child's age of >18 months, using nonpiped water sources, low/normal maternal BMI and no point‐of‐use treatment of drinking water. Finally, underweight was significantly associated with male sex, child's age of >18 months, a cough 2 weeks before the study, absence of a toilet facility and no point‐of‐use drinking water treatment.

Consistent with similar studies, increasing age of the child was negatively associated with wasting and positively associated with stunting (Dake et al., 2019; Garenne et al., 2019; Saaka & Galaa, 2016; Simelane et al., 2020). One of the most striking findings of this study was that the prevalence of all forms of malnutrition (except wasting) appeared to peak at the age range of 54–59 months after reaching its lowest point at 30–41 months. This may be due to decreased attention to older children if the family has another younger child in the household or the fact that existing programmes may prioritize young children due to limited resources. Thus, planners and programme managers should consider the age effect of malnutrition among under‐5 children.

Our study indicated that the proportion of WaSt and wasted children followed a similar pattern with the highest prevalence from 6 to 17 months and a declining prevalence from 18 to 29 months. This is in line with a study in Senegal and a meta‐analysis of 84 countries that found that WaSt declined after 24 months of age (Garenne et al., 2019; Khara et al., 2018). A Ugandan study reported this decline after 36 months (Odei Obeng‐Amoako, et al., 2021). These findings suggest that children aged less than 24 months (maybe up to 36 months) need special attention from families, programmers and the Ministry of Health. Furthermore, it is recommended to establish a system that assesses, manages and reports WaSt like other forms of malnutrition.

Boys had higher odds of wasting, stunting, underweight and WaSt compared with girls in this study (*p* < 0.05). This is consistent with other similar studies for stunting (Blankenship et al., 2020; De Vita et al., 2019; Fenta et al., 2020; Geda et al., 2021; Wali et al., 2020), wasting (Thurstans et al., 2020), underweight (De Vita et al., 2019; Geda et al., 2021) and WaSt (Garenne et al., 2019; Odei Obeng‐Amoako, et al., 2021). Little is known about gender effects on all forms of malnutrition. The difference may be due to hormonal or other unknown factors. This topic calls for further research in order to understand and tackle malnutrition in boys.

Failure to treat drinking water at point of use by common methods in the area such as boiling or chlorination was found to be significantly associated with stunting, underweight and WaSt. This finding is in line with other similar studies (De Vita et al., 2019). However, the presence of diarrhoea was not associated with the outcomes in Kersa. Treatment of water at the point of use was proven to be effective in Pakistan among outpatient severe acute malnutrition (SAM) children by 16.7%–22.2% (Doocy et al., 2018).

Strengths of this study include having a larger sample size that makes the findings more generalizable to the overall Kersa area; double measurement of weight, height and MUAC to reduce measurement bias; and use of ODK open‐source software for collecting, managing and using data to reduce data entry error. However, this study is not without limitations. Some predictors of health conditions such as cough and diarrhoea were self‐reported and may be affected by recall bias.

## 5 CONCLUSION

The prevalence of child wasting, stunting, underweight and WaSt is very high and requires urgent public health intervention in Kersa. Cough is one of the main health‐related factors associated with wasting, underweight and WaSt. Point‐of‐use treatment of drinking water at the household level should be encouraged. Finally, we recommend community‐based programmes for early identification and management of WaSt besides other indicators of malnutrition among 6‐ to 59‐month‐old children.

## CONFLICTS OF INTEREST

The authors declare that they have no conflicts of interest.

## CONTRIBUTIONS

AAR, NA, KT, YD, AT, LB, HE and WF conceived and designed the study. AAR, NA, KT, AT and YD contributed to the acquisition of the data. AAR, NA, HE and WF analysed and interpreted the data. AAR, NA, KT, LB, HE and WF drafted and critically revised the manuscript. All authors have read and approved the final manuscript.

## Data Availability

The data that support the findings of this study are available upon submitting a reasonable request to the corresponding author.
